# Evaluation of Mediastinal Lymphadenopathy in Patients With Non-small Cell Lung Cancer Using Endobronchial Ultrasound-Guided Transbronchial Needle Aspiration

**DOI:** 10.7759/cureus.80973

**Published:** 2025-03-21

**Authors:** Nitesh Goyal, Dibakar Sahu, Sajal De, Ajoy K Behera, Ranganath Ganga, Amit Chowhan

**Affiliations:** 1 Pulmonary, Critical Care, and Sleep Medicine, All India Institute of Medical Sciences, Raipur, IND; 2 Pathology and Laboratory Medicine, All India Institute of Medical Sciences, Raipur, IND

**Keywords:** ebus-tbna, lung cancer staging, mediastinal lymphadenopathy, non-small cell lung cancer, tuberculosis

## Abstract

Introduction

Lung cancer is the leading cause of cancer-related deaths worldwide. Accurate staging of mediastinal lymphadenopathy is crucial, particularly in tuberculosis-endemic regions where benign causes can mimic malignancy. Imaging modalities like computed tomography (CT) and positron emission tomography (PET) scans detect lymph node enlargement but lack specificity. Endobronchial ultrasound-guided transbronchial needle aspiration (EBUS-TBNA) is a minimally invasive technique that enables real-time sampling for histopathological diagnosis. This study evaluates the role of EBUS-TBNA in diagnosing enlarged mediastinal lymph nodes in non-small cell lung cancer (NSCLC) patients and its impact on staging.

Methods

A cross-sectional study was conducted at a tertiary care center in India from May 2023 to August 2024. Thirty NSCLC patients with mediastinal lymphadenopathy (≥10 mm) underwent EBUS-TBNA for histopathological evaluation. Patients with sub-centimetric nodes or severe comorbidities were excluded. CT and PET scans and EBUS findings were analyzed for malignancy, granulomas, or benign conditions.

Results

Among the 30 patients, 25 (83.33%) were male, with a mean age of 59.09±9.95 years. Cough was the most common symptom, affecting 24 patients (80%). A total of 67 lymph nodes were sampled using EBUS-TBNA, revealing malignancy in 57 (85.07%), tuberculosis in two (2.99%), and benign findings in eight (11.94%). Among the 54 18F-fluorodeoxyglucose (FDG)-avid lymph nodes, 44 (81.48%) were malignant, two (3.7%) had tuberculosis, and eight (14.81%) were benign. EBUS-TBNA modified nodal staging in five cases (16.67%), resulting in downstaging for four patients (13.33%) and upstaging for one patient (3.33%). No complications were observed.

Conclusion

EBUS-TBNA is a valuable tool for diagnosing and staging NSCLC, reducing misclassification in tuberculosis-endemic regions. It enhances accuracy by distinguishing malignancy from benign conditions, emphasizing the need for histopathological confirmation. Integrating EBUS-TBNA with imaging can optimize lung cancer management and treatment planning.

## Introduction

Lung cancer is the second most common cause of cancer in India and worldwide [1,2]. It is responsible for the maximum number of cancer-related deaths [1,3]. The outcome of lung cancer depends upon the histopathological type and the stage of the disease [4]. In lung cancer patients, mediastinal lymphadenopathy detected on radiological imaging (lymph node diameter >10 mm in short axis) can change the lung cancer staging entirely and will also affect its management [5].

Mediastinal lymph node enlargement in such patients may not necessarily be due to the involvement of the primary malignancy [5]. Differentials other than the malignant involvement of the enlarged mediastinal lymph nodes in such patients include reactive changes or granulomatous inflammation [5]. The presence of granulomatous inflammation is common in countries with high tuberculosis burden like India, and it can coexist with lung malignancy [6]. Therefore, the etiology of enlarged mediastinal lymph nodes in lung cancer patients should be established to rule out causes other than the malignancy. In patients with lung cancer, the presence of enlarged mediastinal lymphadenopathy due to tuberculosis can downgrade the cancer stage by classifying the lymph nodes as non-malignant. This, in turn, significantly alters the treatment plan and prognosis.

Contrast-enhanced computed tomography (CECT) scan of the chest and 18F-fluorodeoxyglucose-positron emission tomography (FDG-PET) scan are some of the investigations used to detect affected mediastinal lymph nodes [7,8]. However, these tests are not confirmatory due to their limited specificity, as increased metabolic activity on FDG-PET or lymph node enlargement on chest CT is neither diagnostic of malignancy nor indicative of a specific diagnosis. Therefore, it is very necessary to confirm the tissue diagnosis of enlarged mediastinal lymph nodes in patients with lung cancer [9]. Endobronchial ultrasound-guided transbronchial needle aspiration (EBUS-TBNA) is a minimally invasive technique to sample the mediastinal lymph nodes in real time. With this background, the study aims (1) to evaluate the etiology of enlarged mediastinal lymph nodes in patients with non-small cell lung cancer (NSCLC) using EBUS-TBNA, specifically distinguishing between malignant, benign, or tubercular etiology, and (2) to assess the impact of EBUS-TBNA findings on nodal staging in this population. 

## Materials and methods

The study was conducted in the Department of Pulmonary, Critical Care, and Sleep Medicine of All India Institute of Medical Sciences in Raipur, India. Thirty adult patients (>18 years of age) of NSCLC with enlarged mediastinal lymph nodes were included. It was a cross-sectional observational study, and consecutive sampling was done.

Histopathologically diagnosed patients of NSCLC having enlarged (>10 mm in short axis) mediastinal lymph nodes were included. Lung cancer patients with sub-centimetric (<10 mm in short axis) mediastinal lymph nodes, patients with mediastinal lymph nodes that are inaccessible through EBUS, or patients who were not fit for EBUS-TBNA including patients with deranged coagulation profile (INR ≥1.5; platelet count ≤50,000/mm^3^), hemodynamic instability (mean arterial blood pressure ≤65 mmHg), or severe hypoxemia (room air oxygen saturation ≤90%) or patients who were unable to lie supine (patients with orthopnea) were excluded from the study.

The study was conducted from May 2023 to August 2024 after receiving approval from the Institute Ethics Committee of All India Institute of Medical Sciences, Raipur (Chhattisgarh) (approval number: AIIMSRPR/IEC/2023/1326). Informed consent was obtained from all the study participants. After applying inclusion and exclusion criteria, 30 consecutive histopathology-proven NSCLC patients were recruited for the study. All the subjects enrolled were evaluated with detailed history, general physical examination, and systemic examination. CT scan and PET scan (wherever available) findings were noted. EBUS was performed under conscious sedation. Six passes were obtained from each enlarged mediastinal lymph node through the 21-gauge ViziShot EBUS-TBNA needle (Olympus America Inc., Center Valley, Pennsylvania, United States). Rapid on-site evaluation could not be performed for any of the samples obtained. All the enlarged (>10 mm in short axis) mediastinal lymph nodes were sampled. Lymph node aspirate was sent for both cytological (slides) and histopathological (tissue core) examination. The sample was also sent for microscopy examination and Ziehl-Neelsen (ZN) staining, cartridge-based nucleic acid amplification test (CBNAAT), and liquid culture for the diagnosis of tuberculosis. Statistical analysis of data was performed.

Analysis of data

The variables collected were entered in Microsoft Excel (Office 2019) (Microsoft Corporation, Redmond, Washington, United States) and analyzed. The prevalence was reported in proportion. Raw data has been deposited in the institute and will be available from the repository on request.

## Results

After applying exclusion and inclusion criteria, a total of 30 newly diagnosed lung cancer patients were involved in the study. The mean age (mean+standard deviation) of the studied lung cancer patients was 59.09 years (standard deviation 9.95) (Table 1). In the study, 83.33% (n=25) of patients enrolled were males and 16.67% (n=5) were females; 53.33% (n=16) of patients did not have a history of smoking. Cough (n=24; 80%) was the predominant respiratory symptom followed by dyspnea (n=19; 63.33%) and chest pain (n=14; 46.67%). Loss of weight (n=20; 66.67%), loss of appetite (n=18; 60%), and fatigue (n=12; 40%) were the common extrapulmonary symptoms. On examination, nine (30%) patients had clubbing, while superficial cervical lymphadenopathy (cervical) and superior vena cava (SVC) syndrome were found in two patients (6.67%) each.

**Table 1:** Clinical characteristics of the studied lung cancer patients (n=30) SVC: superior vena cava

Clinical characteristics	N (%)
Age+standard deviation (years)	59.09+9.95
Male-to-female ratio	25 (83.33%):5 (16.67%)
Smoker-to-non-smoker ratio	14 (46.67%):16 (53.33%)
Pulmonary symptoms	N (%)
Cough	24 (80%)
Shortness of breath	19 (63.33%)
Chest pain	14 (46.67%)
Sputum	5 (16.67%)
Hemoptysis	4 (13.33%)
Fever	4 (13.33%)
Extrapulmonary symptoms	N (%)
Loss of weight	20 (67.67%)
Loss of appetite	18 (60%)
Fatigue	12 (40%)
Bone pain	5 (16.67%)
Change in voice	3 (10%)
Neck/facial edema	3 (10%)
Headache	2 (6.67%)
Episode of loss of consciousness	1 (3.33%)
No extrapulmonary symptoms	7 (23.33%)
Extrapulmonary findings	N (%)
Clubbing	9 (30%)
Superficial cervical lymphadenopathy	2 (6.67%)
SVC syndrome	2 (6.67%)
Pallor	2 (6.67%)
Vocal cord palsy	1 (3.33%)
No extrapulmonary findings	15 (50%)

Apart from the lung mass and mediastinal lymphadenopathy, CT scans of the studied lung cancer patients showed pleural effusion in 36.67% (n=11) of patients (Table 2). Emphysema, rib erosion, mediastinal vessel involvement in the form of invasion or encasement, and lytic lesions in the vertebrae were found in 6.67% (n=2) of patients each. The size of the lung mass was >3-5 cm in 33.33% (n=10) of patients, while eight (26.67%) patients had lung mass of size >7 cm. CT scan showed that 73.33% (n=22) of patients had enlarged mediastinal lymph nodes at stations 4R and 7. Station 10R and 2R mediastinal lymphadenopathy were found in 23.33% (n=7) and 20% (n=6) of patients, respectively. Adenocarcinoma was the most common histopathological subtype (60%; n=18) among the studied lung cancer patients followed by squamous cell lung cancer in the rest of the patients.

**Table 2:** Radiological characteristics of the studied lung cancer patients (n=30) R: right; L: left

CT scan findings	N (%)
Pleural effusion	11 (36.67%)
Emphysema	2 (6.67%)
Rib erosion	2 (6.67%)
Vascular invasion/encasement	2 (6.67%)
Vertebral lytic lesions	2 (6.67%)
Pericardial effusion	1 (3.33%)
Size of lung mass	N (%)
<3 cm	6 (20%)
>3-5 cm	10 (33.33%)
>5-7 cm	6 (20%)
>7 cm	8 (26.67%)
Enlarged mediastinal lymph node stations	N (%)
2R	6 (20%)
2L	1 (3.33%)
4R	22 (73.33%)
4L	4 (13.33%)
7	22 (73.33%)
10R	7 (23.33%)
10L	4 (13.33%)
11R	1 (3.33%)

PET scans could be done in 25 out of 30 recruited lung cancer patients. Due to financial constraints, the rest of the five patients could not undergo PET-CT, and distant metastases were looked at with the help of CECT of the thorax, abdomen, and pelvis. It showed that 88% (n=22) and 76% (n=19) of patients had FDG-avid mediastinal lymph nodes at station 4R and station 7, respectively. FDG-avid mediastinal lymph nodes at stations 2R and 10R were found in 68% (n=17) and 60% (n=15) of patients, respectively.

In 30 patients, a total of 67 mediastinal lymph nodes were sampled through EBUS (Table 3). Pathological examination of 57 (85.07%) lymph nodes was diagnostic of malignancy. Two (2.99%) lymph nodes among 67 sampled lymph nodes in lung cancer patients showed granuloma and positive ZN stain for acid-fast bacilli (AFB). Eight (11.94%) lymph nodes were negative for both malignancy and granuloma. None of the patients had any complications related to EBUS-TBNA of mediastinal lymph nodes.

**Table 3:** EBUS-TBNA characteristics of the mediastinal lymph nodes in the studied lung cancer patients (n=30) CT: computed tomography; ZN: Ziehl-Neelsen; AFB: acid-fast bacilli; FDG: 18F-fluorodeoxyglucose; EBUS-TBNA: endobronchial ultrasound-guided transbronchial needle aspiration

Complications	N (%)
No complication	30 (100%)
Hemoptysis	0 (0%)
Fever	0 (0%)
Pathological finding of sampled mediastinal lymph nodes enlarged on CT of the chest (n=67)	N (%)
Malignancy	57 (85.07%)
Granuloma and positive ZN stain for AFB	2 (2.99%)
Negative for malignancy and granuloma	8 (11.94%)
Pathological finding of sampled FDG-avid mediastinal lymph nodes (n=54)	N (%)
Malignancy	44 (81.48%)
Granuloma and positive ZN stain for AFB	2 (3.70%)
Negative for malignancy and granuloma	8 (14.81%)
Pathological finding of sampled FDG-non-avid mediastinal lymph nodes (n=5)	N (%)
Malignancy	5 (100%)
Granuloma and positive ZN stain for AFB	0 (0%)
Negative for malignancy and granuloma	0 (0%)

A total of 59 mediastinal lymph nodes were sampled from 25 patients with available PET scans. Out of these, five (8.47%) mediastinal lymph nodes were non-FDG avid on PET scan, but these lymph nodes were sampled through EBUS-TBNA since their short-axis diameter was more than 10 mm. Out of the total 54 FDG-avid mediastinal lymph nodes that were sampled using EBUS-TBNA, 44 (81.48%) were malignant, while eight (14.81%) were negative for malignancy and granuloma. Two (3.7%) of the FDG-avid sampled mediastinal lymph nodes showed granuloma on pathological examination and were also positive for AFB on ZN stain. Out of a total of five FDG-non-avid mediastinal lymph nodes that were sampled using EBUS-TBNA, all (n=5; 100%) were malignant (Table 3). The nodal staging was downgraded in 13.33% (n=4) of patients (from N3 to N0 and N2 to N0 in two patients each), and there was an increase in nodal staging from N2 to N3 in 3.33% (n=1) of patients after EBUS-TBNA-guided pathological examination of the mediastinal lymph nodes (Table 4).

**Table 4:** Change in lymph node staging with EBUS-TBNA in the studied lung cancer patients (n=30) EBUS-TBNA: endobronchial ultrasound-guided transbronchial needle aspiration

Change in lymph node staging	N (%)
No change	25 (83.33%)
N3-N0	2 (6.67%)
N2-N0	2 (6.67%)
N2-N3	1 (3.33%)

## Discussion

Establishing proper techniques is necessary for the accurate diagnosis and staging of NSCLC [4]. Evaluation of mediastinal lymphadenopathy is a vital process in staging lung cancer. In tuberculosis-endemic countries like India, however, enlargement of lymph nodes in lung cancer patients may be due not only to malignancy but also to benign etiologies such as tuberculosis [6]. Thus, the diagnosis is often challenging given that most often the radiological imaging techniques are not that specific, such as CT or PET, in establishing the differential diagnosis between the malignant and non-malignant causes of lymphadenopathy. Thus, the histopathological diagnosis of enlarged mediastinal lymph nodes through EBUS-TBNA is important in ensuring proper treatment as well as accurate staging with minimal misdiagnosis [9]. Our study comprised 30 patients suffering from NSCLC and evaluated the effectiveness of EBUS-TBNA in diagnosing mediastinal lymphadenopathy, especially in a region endemic to tuberculosis.

Diagnostic yield of EBUS-TBNA in lung cancer staging

Our study found that diagnosis (whether malignant or benign etiology) was established in all the sampled mediastinal lymph nodes through EBUS-TBNA. This result was consistent with that obtained by Yasufuku et al. [4], where they stated that a confirmed diagnosis was made in all the mediastinal lymph nodes sampled through EBUS-TBNA. They also compared EBUS-TBNA and mediastinoscopy and showed that it was equal to mediastinoscopy for the detection of malignancy but was much less invasive with minimal complications. The findings of our study also align with those of Lilo et al. [5], where they found that not all FDG-avid mediastinal nodes were malignant (41.9%), thus confirming the use of PET imaging alone to be inadequate for the diagnosis of mediastinal metastasis.

Our study showed that 100% of the enlarged FDG-non-avid mediastinal lymph nodes were malignant on EBUS-TBNA-guided pathological examination. This finding further questions the relevance of PET scans in the nodal staging of lung cancer. If pathological examination had not been carried out for these malignant FDG-non-avid mediastinal lymph nodes, they would have been designated as non-malignant. From the FDG-avid lymph nodes, 14.81% had no malignancy and no granuloma, while 3.7% revealed granuloma with positive AFB staining that indicated tuberculosis. These findings highlight the utility of EBUS-TBNA in the diagnostic process for the malignant as well as non-malignant causes of lymphadenopathy among patients with NSCLC.

Tuberculosis and granulomatous inflammation

Granulomatous inflammation is very common and quite often related to tuberculosis; it is one of the major pitfalls in lung cancer diagnosis in a tuberculosis-endemic region as it tends to cause misinterpretation of enlarged mediastinal lymph nodes as metastatic disease. In our study, based on granuloma and positive ZN staining for AFB, 3.7% of the patients with FDG-avid lymph nodes were diagnosed with tuberculosis, reinforcing prior findings by Pieterman et al. [9] who noted that even though PET imaging depicts metabolically active lymph nodes with high sensitivity, it is not specific enough to distinguish between the malignant and non-malignant causes of lymphadenopathy. Similarly, observations of our study concur with Gould et al. [8], who noted that despite the resolution power of CT and PET scans, their specificity regarding mediastinal metastasis is limited and there seems to be an increasing need for the pathological examination of mediastinal lymph nodes for evaluating benign diseases such as tuberculosis.

Refining cancer staging with EBUS-TBNA

One of the study's most important findings is the downstaging of lymph nodes by EBUS-TBNA in patients with NSCLC. In 13.33% of the cases, EBUS-TBNA downstaged the lymph node (in 6.67% N3 shifted to N0 and in 6.67% N2 to N0). These findings have not changed the staging of lung cancer in the studied subjects, since all of them were already stage IV disease and a mere change in nodal stage would not change the overall lung cancer staging. But had any such patient presented at an early stage, a change in nodal staging would have created a significant impact on the lung cancer staging. A second benefit that EBUS-TBNA provides is the proper determination of the extent of lymph node involvement, thereby preventing the overtreatment of a significant percentage of patients who truly have lymphadenopathy due to benign or non-malignant reasons such as tuberculosis.

Several studies also documented that EBUS-TBNA can significantly change the cancer staging. Yasufuku et al. [10] noticed that the EBUS-TBNA was equivalent to mediastinoscopy in staging the mediastinal lymph nodes and, in some cases, it caused downstaging by revealing a status of non-malignant lymphadenopathy. Likewise, Cerfolio et al. [11] highlighted the value of EBUS-TBNA in the confirmation or exclusion of metastatic disease, and it may also negate the need for unnecessary treatment including surgeries in those patients having benign lymphadenopathy.

Comparison with mediastinoscopy

For a long time, mediastinoscopy has been regarded as the gold standard for the diagnosis and staging of mediastinal lymph nodes. However, in recent times, several studies have shown that one may obtain nearly the same results with EBUS-TBNA. Our study also shows that in 83.33% of patients, the pre- and post-procedure lymph node staging was concordant, and in a very few, it did show variation. This high concordance rate reflects the diagnostic accuracy of EBUS-TBNA for staging mediastinal lymph nodes when compared with imaging modalities. Ernst et al. [12] demonstrated that the sensitivity of EBUS-TBNA was 87% and its negative predictive value was 78%, which were both higher than mediastinoscopy where a sensitivity of 68% and a negative predictive value of 59% were documented.

Similarly, Yasufuku et al. [10] demonstrated that EBUS-TBNA was equally effective to mediastinoscopy in diagnosing and staging mediastinal lymph nodes but offered a much less invasive approach with minimal complication rate. The above findings were in line with our study where a very high diagnostic yield for EBUS-TBNA was seen and no patient had any significant complications.

Limitations of our study

Although our study has brought forth very notable findings, there are limitations to this study. Firstly, the sample size was only 30 patients, which makes our study less generalizable. More importantly, although our results accord well with those in larger studies, a larger sample size and a more heterogeneous population would support even stronger data and permit further appropriately detailed diagnostic efficacy of EBUS-TBNA. Secondly, the study has been conducted in an institution where most of the patients presented in an advanced stage of disease, resulting in no overall change in the cancer stage even after a decrease in nodal stage in such patients. If the present study can be replicated in patients in an early stage of lung cancer, the results would impart more significant information. Lastly, even till date, mediastinoscopy is the gold standard test for the evaluation of mediastinal lymphadenopathy in lung cancer patients. Hence, the results of EBUS-TBNA should have been compared with mediastinoscopy, but it was beyond the scope of the current study. This counts towards another limitation of the study.

Clinical implications

The results of our study have important clinical implications with respect to managing patients diagnosed with NSCLC in countries where tuberculosis is endemic. Thus, EBUS-TBNA is critical for the accurate staging of lung cancer in confirming or excluding malignancy within mediastinal lymph nodes, which further guides treatment and improves outcomes. In areas with a higher prevalence of tuberculosis, EBUS-TBNA also helps to differentiate between the malignant and non-malignant causes of lymphadenopathy, thereby avoiding overtreatment or unnecessary surgeries in lung cancer patients.

## Conclusions

This study adds to the growing body of evidence supporting the use of EBUS-TBNA for diagnosing and staging mediastinal lymphadenopathy in NSCLC patients. By confirming the presence of malignancy in FDG-avid lymph nodes and downstaging lymph node involvement in a significant number of patients, EBUS-TBNA demonstrated its value in improving diagnostic accuracy and guiding treatment decisions. The findings are particularly relevant in tuberculosis-endemic regions, where EBUS-TBNA can help differentiate between the malignant and benign causes of lymphadenopathy, preventing overtreatment and improving patient outcomes. As the role of EBUS-TBNA continues to expand, future research should focus on optimizing its use in combination with other diagnostic modalities to further enhance its diagnostic efficacy.
